# Alternative splicing and nonsense-mediated decay of circadian clock genes under environmental stress conditions in *Arabidopsis*

**DOI:** 10.1186/1471-2229-14-136

**Published:** 2014-05-19

**Authors:** Young-Ju Kwon, Mi-Jeong Park, Sang-Gyu Kim, Ian T Baldwin, Chung-Mo Park

**Affiliations:** 1 Department of Chemistry, Seoul National University, Seoul 151-742, Korea; 2 Department of Molecular Ecology, Max Planck Institute for Chemical Ecology, 07745 Jena, Germany

**Keywords:** *Arabidopsis thaliana*, Circadian clock, Transcription factor, Alternative splicing, Nonsense-mediated decay (NMD), Environmental stress

## Abstract

**Background:**

The circadian clock enables living organisms to anticipate recurring daily and seasonal fluctuations in their growth habitats and synchronize their biology to the environmental cycle. The plant circadian clock consists of multiple transcription-translation feedback loops that are entrained by environmental signals, such as light and temperature. In recent years, alternative splicing emerges as an important molecular mechanism that modulates the clock function in plants. Several clock genes are known to undergo alternative splicing in response to changes in environmental conditions, suggesting that the clock function is intimately associated with environmental responses via the alternative splicing of the clock genes. However, the alternative splicing events of the clock genes have not been studied at the molecular level.

**Results:**

We systematically examined whether major clock genes undergo alternative splicing under various environmental conditions in *Arabidopsis*. We also investigated the fates of the RNA splice variants of the clock genes. It was found that the clock genes, including *EARLY FLOWERING 3* (*ELF3*) and *ZEITLUPE* (*ZTL*) that have not been studied in terms of alternative splicing, undergo extensive alternative splicing through diverse modes of splicing events, such as intron retention, exon skipping, and selection of alternative 5′ splice site. Their alternative splicing patterns were differentially influenced by changes in photoperiod, temperature extremes, and salt stress. Notably, the RNA splice variants of *TIMING OF CAB EXPRESSION 1* (*TOC1*) and *ELF3* were degraded through the nonsense-mediated decay (NMD) pathway, whereas those of other clock genes were insensitive to NMD.

**Conclusion:**

Taken together, our observations demonstrate that the major clock genes examined undergo extensive alternative splicing under various environmental conditions, suggesting that alternative splicing is a molecular scheme that underlies the linkage between the clock and environmental stress adaptation in plants. It is also envisioned that alternative splicing of the clock genes plays more complex roles than previously expected.

## Background

The circadian clock is an endogenous time-keeping system that coordinates the physiology and behavior of a living organism to its environment [1]. In plants, the clock modulates rhythmic leaf movement, elongation rate of hypocotyls, roots, and stems, stomata aperture, stem circumnutation, and flower opening [1,2].

Three major interlocked feedback loops constitute the plant circadian clock: the central loop, the morning loop, and the evening loop [3-5]. The central loop is mediated by the reciprocal repression between the morning-phased MYB transcription factors, CIRCADIAN CLOCK ASSOCIATED 1 (CCA1) and LATE ELONGATED HYPOCOTYL (LHY), and the evening-phased pseudo-response regulator TIMING OF CAB EXPRESSION 1 (TOC1) [6,7]. In the morning loop, CCA1 and LHY promote the transcription of *PSEUDO*-*RESPONSE REGULATOR 9* (*PRR9*) and *PRR7* genes [8,9]. Closing the loop, the PRR members inhibit the transcription of *CCA1* and *LHY* genes by sequentially binding to the gene promoters from early morning (PRR9) through mid-day (PRR7) to evening (PRR5) [10,11]. The evening loop is illustrated by TOC1 and a hypothetical component Y, the expression of which is repressed by TOC1 and, in turn, activates *TOC1* expression [12]. Recent studies have shown that three evening-phased factors, EARLY FLOWERING 3 (ELF3), ELF4, and LUX ARRHYTHMO (LUX), form the EVENING COMPLEX (EC), which represses *PRR9* gene and *LUX* gene itself [13,14], indicating that the auto-inhibition of EC replaces the component Y in the evening loop [15].

The circadian system is substantially influenced by external cues. Phytochrome- and cryptochrome-mediated light signals mediate the induction of *CCA1*, *LHY*, and *PRR9* genes [8,16,17]. Temperatures also affect the amplitudes and rhythms of the clock gene expression [18]. In addition, growth hormones and abiotic stresses modulate the clock function. It has been observed that accumulation of *CCA1*, *TOC1*, and *GIGANTEA* (*GI*) gene transcripts is differentially regulated by abscisic acid, brassinosteroid, and auxin [19]. High light stress induces *CCA1* gene [20], linking the clock with plant stress adaptation.

The clock components are also regulated at the posttranscriptional and protein levels. It has been shown that the stability of *CCA1* mRNA and the translation of *LHY* mRNA are influenced by light [21,22]. In addition, the F-box protein ZEITLUPE (ZTL) is responsible for the dark-induced degradation of TOC1 protein [23]. Furthermore, temperature-dependent phosphorylation of CCA1 modulates its binding to target gene promoters [24]. Most recently, chromatin remodeling and alternative splicing of the clock genes have been described as fundamental processes in the regulation of the clock function [25].

Some of the clock genes have been shown to undergo alternative splicing in response to abiotic stresses in plants [26,27], among which temperature regulation of *CCA1* alternative splicing is best characterized. *CCA1* alternative splicing produces two protein isoforms, the full-size CCA1α form and the truncated CCA1β form that lacks the MYB DNA-binding motif [27]. CCA1β competitively inhibits CCA1α activity by forming nonfunctional heterodimers that are excluded from DNA binding. *CCA1* alternative splicing is suppressed by low temperatures. Under low temperature conditions, CCA1β production is reduced, and thus CCA1α activity is elevated, leading to the stimulation of freezing tolerance [27], linking the clock with temperature response.

Recently, it has been reported that alternatively spliced RNA isoforms of some clock genes are degraded through the nonsense-mediated decay (NMD) pathway [28-33], unlike the productive alternative splicing of *CCA1* gene. NMD has evolved as an mRNA quality control mechanism that degrades mRNA molecules harboring premature termination codons (PTCs), which generate truncated proteins that are harmful to cellular energy metabolism, and those having aberrantly long 3′ untranslated regions (3′-UTRs) [32,33]. It is thus possible that alternative splicing serves as a precise mechanism for controlling the mRNA levels of the clock genes, depending on endogenous and external conditions.

In this study, we systematically investigated the alternative splicing patterns of major clock genes under various environmental conditions. We also examined the fates of the RNA splice variants. Our study shows that alternative splicing of the clock genes is differentially influenced by photoperiod and a variety of abiotic stresses. The results of our study show that although RNA splice variants of some clock genes are predicted to encode truncated versions of the authentic proteins, those of other clock genes do not appear to encode specific proteins and, instead, are degraded through the NMD pathway. It is envisioned that alternative splicing plays more complex roles in the clock function than previously expected.

## Results

### Major clock genes undergo extensive alternative splicing

On the basis of the prevalence of alternative splicing events in the plant circadian clock genes in the literature [20,26,27,34,35], we anticipated that alternative splicing of the core clock genes constitutes a critical component of the clock function. Previous reports have shown that alternative splicing of *CCA1* is suppressed by low temperatures [20,27,35]. The alternative protein isoform (CCA1β), which lacks the protein domain required for DNA binding, acts as a dominant negative regulator of the authentic CCA1 transcription factor (CCA1α), thus providing a self-regulatory circuit that links the clock with temperature stress response.

To extend our understanding of the functional relationship between the clock genes and environmental stress responses, we selected a group of major clock genes that constitutes the plant circadian clock and investigated whether these undergo alternative splicing and their alternative splicing patterns are altered under environmental stress conditions.

**Figure 1:**
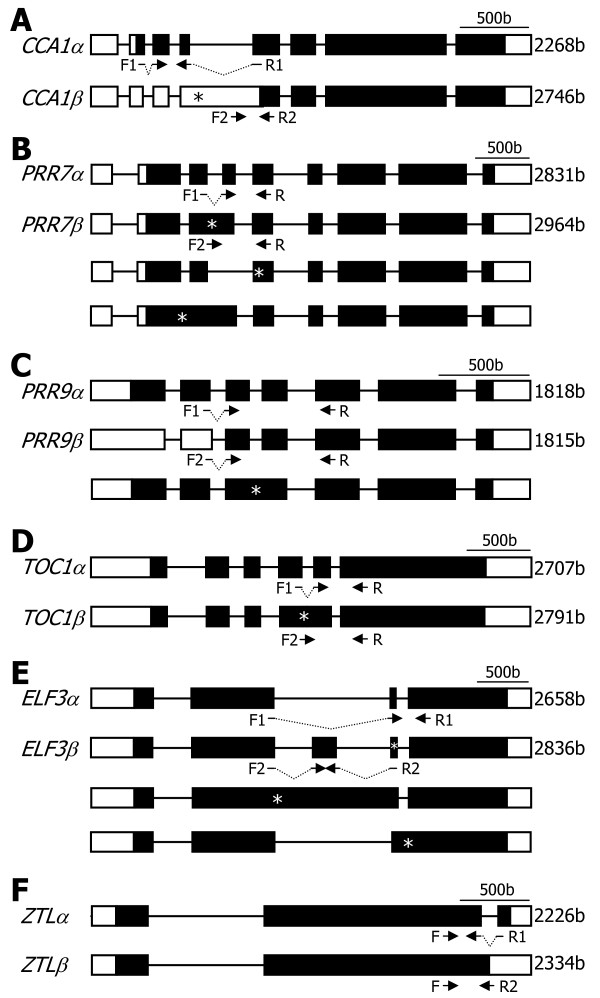
**Genomic structures of major clock genes.** The clock gene sequences were analyzed using the softwares provided by the TAIR database. The predicted genome structures of *CCA1***(A)**, *PRR7***(B)**, *PRR9***(C)**, *TOC1***(D)**, *ELF3***(E)**, and *ZTL***(F)** genes are displayed. Black boxes depict exons. White boxes are 5′ and 3′ UTRs. F and R are primers used for RT-PCR analysis of the RNA splice variants (see Figure 2). The *α* transcripts encode full-size, authentic proteins, and the *β* transcripts encode truncated forms. Asterisks indicate premature termination codons (PTCs). b, bases. The genomic structure of *CCA1* gene, which has already been reported by us [27], was included here for the benefit of the reader.

Analysis of gene structures deposited in the public databases and literature search revealed that *PRR7*, *PRR9*, *TOC1*, and *ZTL* genes as well as *CCA1* gene undergo alternative splicing [26,27,34,35], each producing two or more RNA splice variants (Figure 1). For each clock gene, the *α* transcript represents the RNA splice variant that retains all the exons but do not have any introns. The *β* transcript represents the one that exists at the highest level among the RNA splice variants other than the *α* transcript.

*CCA1* alternative splicing is mediated by the retention of intron 4 and introduces a PTC into *CCA1β* transcript (Figure 1A). *PRR7* alternative splicing is somewhat complicated. It is mostly mediated by the retention of intron 3, resulting in *PRR7β* transcript (Figure 1B and Additional file 1). A PTC is introduced into the *PRR7β* transcript. Notably, it is also mediated by the skipping of exon 4 and the retention of introns 2 and 3 [26,34,35]. *PRR9* alternative splicing is unique, among others, in that the major alternatively spliced variant (*PRR9β*) is produced by selection of alternative 5′ splice site in intron 2 (Figure 1C and Additional file 2). The presence of two additional RNA splice variants has also been recently reported [26,34,35].

A single *TOC1* cDNA sequence was identified in the TAIR database. However, it has been shown that an alternative splicing event occurs by the retention of intron 4 [26,34], introducing a PTC into *TOC1β* transcript (Figure 1D and Additional file 3). It has been reported that RNA splice variants of *ELF3* gene are hardly detected in wild-type plants, but several RNA splice variants are detected in the *skip-1* mutant, which is defective in its splicing machinery [34], possibly due to the retention of intron 2 or 3 (Figure 1E). We found that the *ELF3* gene undergoes alternative splicing in wild-type plants (Additional file 4). In addition, it was found that the *ELF3β* transcript is derived from the inclusion of a new alternative exon and a PTC is introduced into the splice variant.

There are two *ZTL*-specific cDNA sequences (*ZTLα* and *ZTLβ*) in the public database. Sequence comparison and direct sequencing of RT-PCR products revealed that the *ZTL* alternative splicing is mediated by the retention of intron 2 (Figure 1F and Additional file 5). The *ZTLβ*-encoded protein has been considered as an authentic ZTL enzyme in the literature [23], which is probably because the abundance of the *ZTLβ* transcript is much higher than that of the *ZTLα* transcript (see below).

### The modes of splicing events are diverse in the clock genes

**Figure 2:**
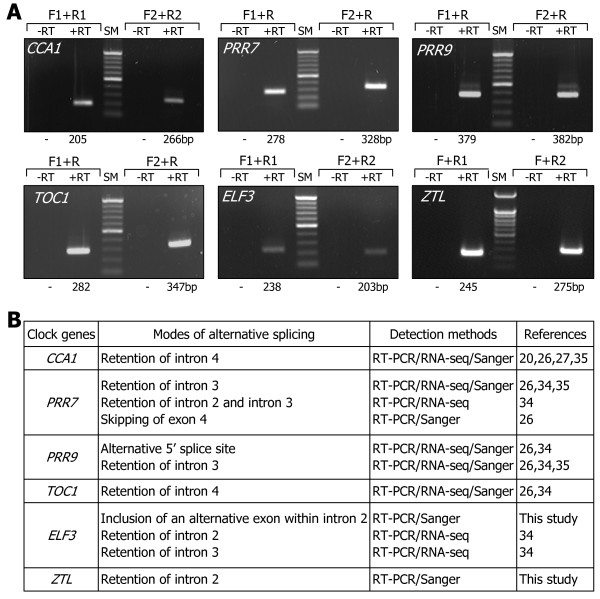
**Detection of RNA splice variants of the clock genes. A**. Detection of RNA splice variants by RT-PCR. Total RNA samples were isolated from 10-day-old Col-0 plants grown on MS-agar plates under LDs at peak ZT point for each clock gene and subject to RT-PCR. Gene-specific F and R primer sets, as indicated in Figure 1, were used to detect the transcript isoforms of each clock gene. PCR reactions were also performed without reverse transcription (-RT) to verify the lack of genomic DNA contamination. The sizes of the PCR products are provided at the bottom of the figure. SM, DNA size marker. bp, base pair. **B**. Modes of splicing events. Detection methods for the alternative splicing events are listed in the 3^rd^ column with the references indicated in the 4^th^ column. The nucleotide sequences of the RNA splice variants were determined (This work) or verified by direct DNA sequencing in this work. RNA-seq, RNA sequencing. Sanger, DNA sequencing by Sanger method.

The abundances of RNA splice variants other than *α* and *β* transcripts were relatively very low in most cases ([26,34,35], this study). We therefore decided to further investigate only the *α* and *β* transcripts for each clock gene. The predicted alternative splicing modes of the clock genes were verified by cloning of the RNA splice variants by RT-PCR and direct DNA sequencing (Additional files 1, 2, 3, 4, and 5). Total RNA samples were subjected to RT-PCR using primer pairs that are specific to each RNA splice variant. The results showed that all the RT-PCR products have the sizes that were inferred from the predicted alternative splicing modes of the clock genes (Figure 2A). No RT-PCR products were detected when reverse transcription was omitted prior to PCR amplifications, indicating that total RNA samples used were not contaminated with genomic DNA.

The modes of alternative splicing are diverse in the clock genes (Figure 2B). Retention of specific introns mediates the alternative splicing of *CCA1*, *PRR7*, *TOC1*, *ZTL*, and *ELF3* genes. Exon skipping is involved in *PRR7* alternative splicing. Meanwhile, alternative 5′ splice site contributes to *PRR9* alternative splicing. Alternative splicing of *ELF3* gene was the most complicated. Retention of intron 2 or 3 has been implicated in the *ELF3* alternative splicing [34]. However, direct sequencing of PCR products revealed that an additional RNA splice variant (*ELF3β*), which is probably most abundant among the splice variants, was produced by the inclusion of an alternative exon.

**Figure 3:**
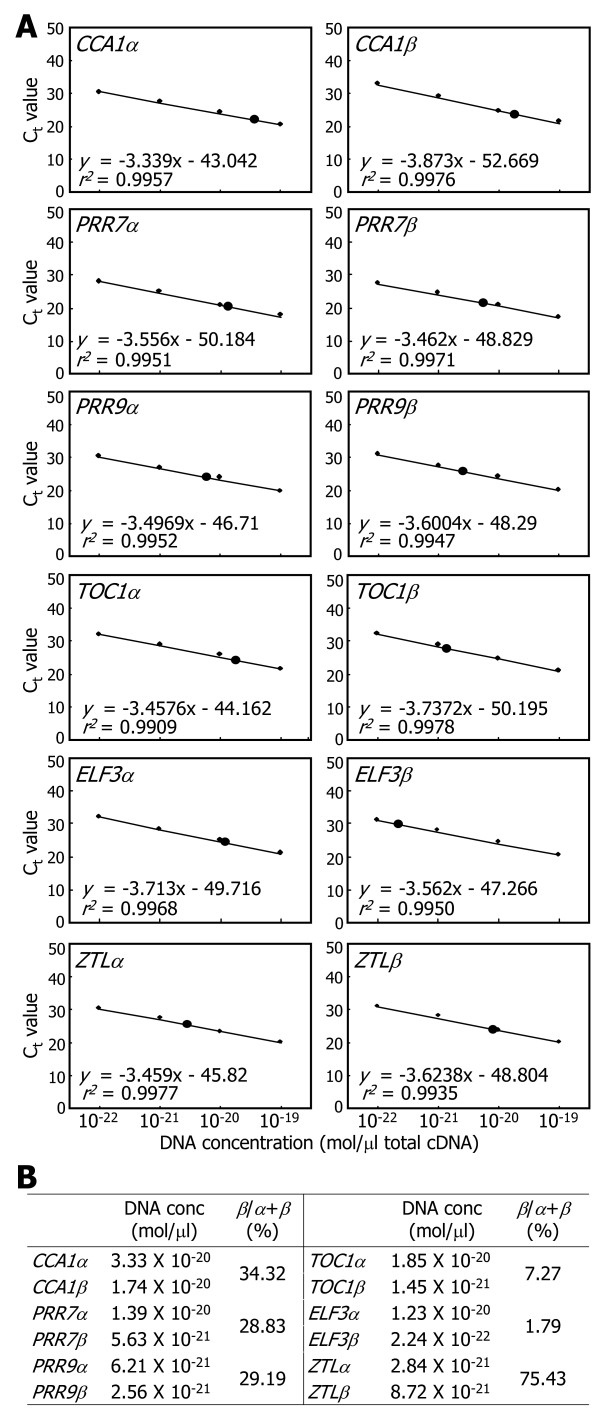
**Absolute quantification of alternatively spliced RNA variants.** Ten-day-old Col-0 plants grown on MS-agar plates were used for the extraction of total RNA samples. To maximize the sensitivity of detection, plants were harvested at the phase of peak expression for each gene. A series of 10-fold dilutions of plasmid DNA containing each gene sequence was used to generate a standard curve. The regression line from the dilution curve was used to determine the concentration of each RNA splice variant. Black circles represent the absolute amounts of RNA splice variants **(A)**. C_T_, threshold cycle. The percentages of *β*/*α* + *β* were calculated for each clock gene **(B)**.

We measured the absolute amounts of the RNA splice variants of each clock gene by qRT-PCR analysis (Figure 3A), as has been described previously [36,37]. Ten-day-old plants grown on MS-agar plates under long days (LDs, 16-h light and 8-h dark) were harvested at zeitgeber time (ZT) points of peak expression for individual clock genes (e.g. ZT0 for *CCA1* and *ZTL*, ZT8 for *PRR9*, ZT4 for *PRR7*, and ZT12 for *TOC1* and *ELF3*), thereby maximizing the detection sensitivity of a small quantity of mRNA. Absolute quantitation of the α and β RNA splice variants of each clock gene showed that the ratios (%) of β/α + β were variable among them (Figure 3B). The ratio of *CCA1* RNA splice variants was 34.32%, similar to what has been previously reported [27]. Those of the RNA splice variants of *PRR7* and *PRR9* genes were approximately 29%. In contrast, those of *TOC1* and *ELF3* genes were relative low (<10%). One distinction was *ZTL* gene. The *β* transcript level was higher than that of the *α* transcript (*β*/*α* + *β* = ~75%), which was in contrast to the other clock genes. It is assumed that the physiological significance of alternative splicing varies in each clock gene.

### Some RNA splice variants of the clock genes are degraded by NMD

Alternatively spliced RNA variants containing a PTC enter either the productive or unproductive pathway. In the productive pathway, the mRNA is translated into a protein that is structurally distinct from the authentic protein. One example is the alternative splicing of *CCA1*, in which the CCA1β protein isoform possesses protein domains required for dimer formation and transcriptional activation but lacks the MYB DNA-binding domain [27]. In contrast, in the unproductive pathway, the transcript is degraded via the NMD-mediated degradation pathway [30].

**Figure 4:**
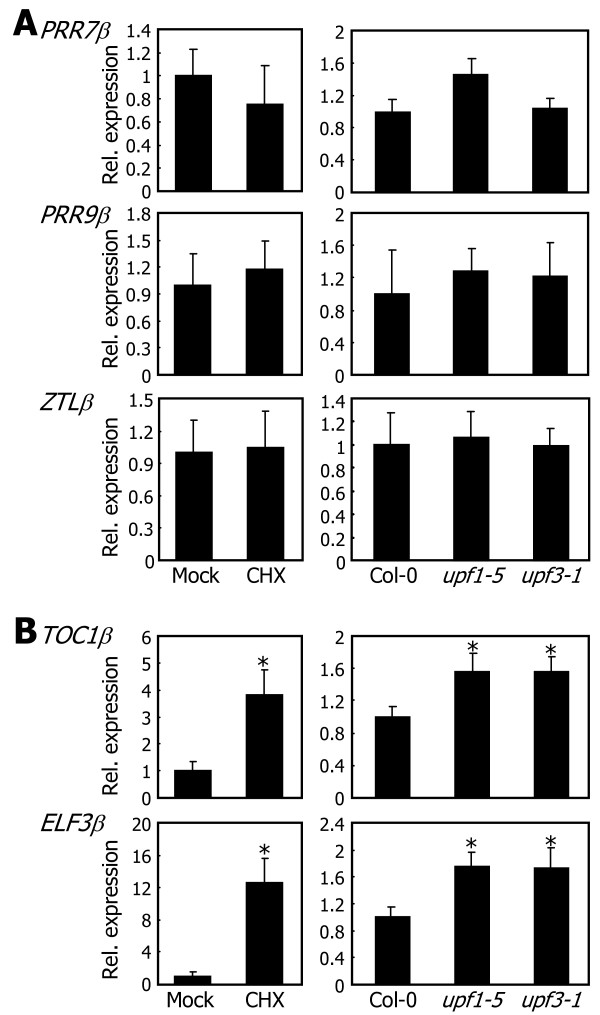
**The fates of RNA splice variants.** Steady-state levels of the *β* transcripts were determined by qRT-PCR in Col-0 plants after CHX treatments (left panels) and in the *upf1*-*5* and *upf3*-*1* mutants (right panels). Biological triplicates were averaged and statistically treated using Student *t*-test (*P < 0.01). Bars indicate standard error of the mean. **A**. The fates of *PRR7β*, *PRR9β*, and *ZTLβ* transcripts. **B**. The fates of *TOC1β* and *ELF3β* transcripts.

To investigate the fates of the RNA splice variants of the clock genes, we employed two assay systems: cycloheximide (CHX) treatment and NMD-defective *Arabidopsis* mutants that are routinely employed for this purpose in the literature. NMD requires translation, and thus the translational inhibitor CHX suppresses the NMD-mediated degradation of mRNA [38,39]. Wild-type *Arabidopsis* plants were treated with CHX, and the levels of *β* transcripts were determined by qRT-PCR. It was found that whereas the levels of *PRR7β*, *PRR9β*, and *ZTLβ* transcripts were not influenced by CHX treatments (Figure 4A, left panels), those of *TOC1β* and *ELF3β* transcripts were significantly elevated after CHX treatments (Figure 4B, left panels).

We next examined the *β* transcript levels of each clock gene in the *upf1-5* and *upf3-1 Arabidopsis* mutants, in which the NMD pathway is impaired [40,41]. The levels of *PRR7β*, *PRR9β*, and *ZTLβ* transcripts in the mutants were comparable to those in wild-type plants (Figure 4A, right panels). In contrast, those of *TOC1β* and *ELF3β* transcripts were higher by approximately two-fold in the mutants than in wild-type plants (Figure 4B, right panels). We also examined the levels of *TOC1β* and *ELF3β* transcripts in the *upf1-5* and *upf3-1* mutants under heat conditions. The *β* transcript levels were even higher in the mutants than in wild type plants when grown at 37°C (Additional file 6). The more prominent differences in the *TOC1β* and *ELF3β* transcript levels at 37°C is due to the heat-induced alternative splicing of *TOC1* and *ELF3* genes (see below). Based on these observations, it was concluded that whereas the *PRR7β*, *PRR9β*, and *ZTLβ* transcripts are likely to encode specific proteins, like the *CCA1β* transcript [27], the *TOC1β* and *ELF3β* transcripts are probably targeted by NMD. The sensitivity of the *TOC1β* and *ELF3β* transcripts to NMD is also consistent with the notion that the steady-state levels of NMD target mRNAs were relatively low in many cases [30,42,43].

Since the identities of *ZTLα* and *ZTLβ* transcripts are currently unclear ([23], this work), we also examined the effects of CHX and *upf1-5* and *upf3-1* mutations on the accumulation of *ZTLα* transcript. We found that the *ZTLα* transcript level was not affected by CHX treatments (Additional file 7). It was also unaltered in the *upf1-5* and *upf3-1* mutants, like that of *ZTLβ* transcript under identical assay conditions. It is therefore likely that the *ZTLα* transcript is not targeted by NMD and, instead, encodes a distinct protein, like the *ZTLβ* transcript.

### Protein isoforms of the clock components are defective in different functional domains

Some RNA splice variants, such as *PRR7β*, *PRR9β*, and *ZTLβ* transcripts that are insensitive to NMD, were predicted to encode truncated proteins that harbor altered protein structural organizations, as has been demonstrated with CCA1β protein isoform [27]. In many cases, these structural alterations in the truncated forms include deletions, insertions, or substitutions of certain protein domains [44].

We analyzed the structural organization of the predicted protein isoforms of CCA1, PRR7, PRR9, and ZTL using the analysis tools in the SMART and Pfam databases (http://smart.embl-heidelberg.de/ and http://pfam.sanger.ac.uk/, respectively). The amino acid sequences of the protein isoforms were obtained either from the TAIR database or deduced from the nucleotide sequences of RT-PCR products.

**Figure 5:**
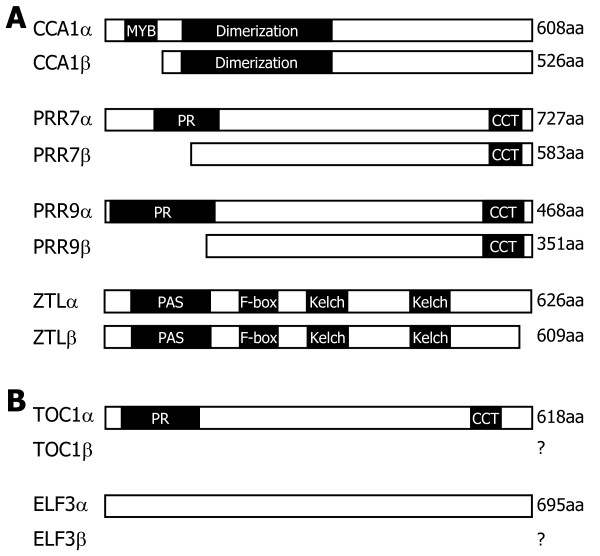
**Domain structures of alternatively spliced protein isoforms.** The protein domain structures were analyzed using the SMART and Pfam databases. Black boxes indicate the conserved protein domains. PR, pseudo-receiver; CCT, CONSTANS, CONSTANS-LIKE, and TOC1; PAS, Per-ARNT-Sim; aa, amino acid. **A**. Protein domain structures of CCA1, PRR7, PRR9, and ZTL and their protein isoforms. **B**. Protein domain structures of TOC1 and ELF3.

Two possible translation products were deduced from *PRR7β* transcript. One protein isoform would be a truncated form containing the N-terminal pseudo-receiver (PR) domain, which is generated by the translation from the start codon to PTC (Figure 1). In this translation scheme, the *PRR7β* transcript harbors a long 3′-UTR. It has been previously shown that alternatively spliced RNA variants having long 3′-UTRs are frequently targeted by NMD [30]. The other protein isoform is a truncated form lacking the N-terminal PR domain, which was marked as PRR7β (Figure 5A). On the basis of the structural similarity of PRR7β to CCA1β and PRR9β and the insensitivity of *PRR7β* transcript to CHX, we believe that the *PRR7β* transcript encodes the PRR7β protein that harbors the N-terminal truncation.

PRR7β and PRR9β protein isoforms possess the CONSTANS, CONSTANS-LIKE, and TOC1 (CCT) domains but lack the N-terminal PR domain (Figure 5), which mediates interactions with other proteins, such as PRR5 [23,45-47]. The overall structures of the predicted ZTLα and ZTLβ protein isoforms were similar to each other except for the short C-terminal sequence. The ZTLβ isoform is slightly smaller than the ZTLα isoform by 17 residues (Figure 5A). We were unable to identify any distinct protein motifs in the C-terminal region of the ZTL proteins, and thus it is currently unclear whether the two ZTL protein isoforms are functionally distinct or not.

Our data showed that *TOC1β* and *ELF3β* transcripts were targeted by NMD and were not expected to encode any proteins (Figure 5B).

### Short days suppress the alternative splicing of *TOC1* and *ELF3* genes

Plants use the circadian clock to monitor daylength changes in inducing seasonal developmental responses [48,49]. We therefore hypothesized that photoperiod influences the alternative splicing patterns of the clock genes.

**Figure 6:**
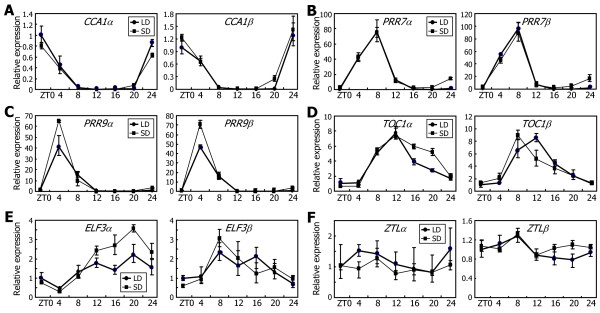
**Effects of photoperiod on the alternative splicing of the clock genes.** Ten-day-old Col-0 plants grown on MS-agar plates under either LDs or SDs were harvested at the indicated ZT points for the extraction of total RNA samples. The levels of the RNA splice variants of *CCA1***(A)**, *PRR7***(B)**, *PRR9***(C)**, *TOC1***(D)**, *ELF3***(E)**, and *ZTL***(F)** genes were determined by qRT-PCR. Biological triplicates were averaged. Bars indicate the standard error of the mean.

*Arabidopsis* plants were entrained to either LDs or short days (SDs, 8-h light and 16-h light), and the levels of alternatively spliced RNA variants were compared by qRT-PCR. The amplitudes and rhythms of *CCA1* and *PRR7* gene expression were not detectably altered under SDs (Figure 6A and B, respectively). Under SDs, *PRR9* gene was induced, but its expression rhythms were maintained (Figure 6C). It seems that the alternative splicing of the morning-phased genes is not discernibly influenced by photoperiod.

We observed that the levels of *TOC1α* and *ELF3α* transcripts were higher under SDs than under LDs, evidently during the night (Figure 6D and E, respectively). In contrast, the levels of *TOC1β* transcript were lower during the night under SDs, and those of *ELF3β* transcript were not altered under identical conditions compared with LDs. Notably, the peak level of the *TOC1β* transcript shifted from ZT12 under LDs to ZT8 under SDs. Together, these observations indicate that SDs suppress the alternative splicing of the *TOC1* and *ELF3* genes. There were no discernible effects of SDs on the alternative splicing of *ZTL* gene (Figure 6F).

### Low temperatures suppress *CCA1* and *ELF3* alternative splicing but induce *TOC1* alternative splicing

Low temperatures dampen the cyclic expression of the clock genes, resulting in the repression of the clock function [18]. Similarly, low temperatures suppress the alternative splicing of *CCA1*, and the imbalance between CCA1α and CCA1β protein isoforms leads to disturbed circadian rhythms and induction of freezing tolerance [27]. It was therefore suspected that low temperatures would also affect the alternative splicing of other clock genes.

**Figure 7:**
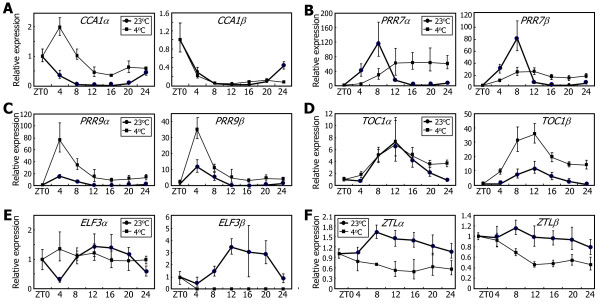
**Effects of low temperatures on the alternative splicing of the clock genes.** Ten-day-old Col-0 plants grown on MS-agar plates under LDs were transferred to 4°C under continuous light conditions. Whole plant materials were harvested at the indicated ZT points. The levels of the RNA splice variants of *CCA1***(A)**, *PRR7***(B)**, *PRR9***(C)**, *TOC1***(D)**, *ELF3***(E)**, and *ZTL***(F)** genes were determined by qRT-PCR. Biological triplicates were averaged. Bars indicate the standard error of the mean.

*Arabidopsis* plants were exposed to 4°C, and the levels of alternatively spliced RNA variants were measured by qRT-PCR. To eliminate the effects of light–dark transitions, the assays were conducted under continuous light conditions. It was found that the rhythmic accumulation patterns of *α* and *β* transcripts of the clock genes were significantly altered at low temperatures. *CCA1* alternative splicing was suppressed at low temperatures; the levels of *CCA1α* transcripts were elevated, whereas those of *CCA1β* transcripts remained low (Figure 7A), as previously described [27].

The levels of both *PRR7α* and *PRR7β* transcripts were lower during the subjective day and higher during the subjective night compared to those at 23°C (Figure 7B), indicating that low temperatures do not affect *PRR7* alternative splicing but diminish its rhythmic expression. PRR9 is functionally redundant with PRR7 [12]. However, the effects of low temperatures on *PRR9* expression were distinct from those on *PRR7* expression. The levels of both *PRR9α* and *PRR9β* transcripts were markedly elevated at low temperatures throughout the time course, and the rhythmic expression was enhanced (Figure 7C), indicating that low temperatures do not affect its alternative splicing.

The effects of low temperatures on the alternative splicing of the evening-phased genes were quite diverse. While the levels of *TOC1α* transcripts remained unchanged, those of *TOC1β* transcripts were markedly higher at low temperatures (Figure 7D), indicating that low temperatures induce *TOC1* alternative splicing. The levels of *ELF3α* transcripts were largely unaffected but loosed rhythmicity at low temperatures (Figure 7E). In contrast, the levels of *ELF3β* transcripts were drastically reduced, showing that *ELF3* alternative splicing is suppressed at low temperatures. *ZTL* expression was suppressed at low temperatures, but its alternative splicing remained unaltered (Figure 7F).

### Heat induces the alternative splicing of *CCA1*, *PRR7*, *TOC1*, and *ELF3* genes

Heat stress has become an important issue in the field because of recent global warming that extensively affects plant growth and distribution [50]. Because the clock is entrained at least in part by temperature, heat would certainly influence the clock function. However, little is known about the relationship between heat stress and the clock. We therefore examined the effects of heat on the alternative splicing of the clock genes. The heat assays were performed under continuous light conditions to eliminate the effects of light–dark transitions.

**Figure 8:**
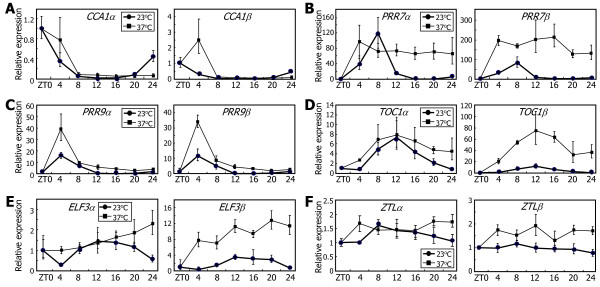
**Effects of heat on the alternative splicing of the clock genes.** Ten-day-old Col-0 plants grown on MS-agar plates under LDs were transferred to 37°C under continuous light conditions. Whole plant materials were harvested at the indicated ZT points. The levels of the RNA splice variants of *CCA1***(A)**, *PRR7***(B)**, *PRR9***(C)**, *TOC1***(D)**, *ELF3***(E)**, and *ZTL***(F)** genes were determined by qRT-PCR. Biological triplicates were averaged. Bars indicate the standard error of the mean. Note that the expression data at 23°C are identical to those in Figure 7.

Interestingly, the balance between *α* and *β* transcripts varied among different clock genes, whereas most clock genes were induced at 37°C. The levels of *CCA1β* and *PRR7β* transcripts were significantly elevated at some ZT points after heat treatments (Figure 8A and B, respectively), showing that their alternative splicing was accordingly induced. The levels of *PRR9α* and *PRR9β* transcripts were elevated to a similar degree, showing that its alternative splicing is not affected by heat (Figure 8C). The elevation of *TOC1β* and *ELF3β* transcript levels were more prominent than that of *TOC1α* and *ELF3α* transcript levels (Figure 8D and E, respectively), suggesting that their alternative splicing was induced by heat. Heat effects were marginal on *ZTL* expression. The levels of both *ZTLα* and *ZTLβ* transcripts were slightly elevated after heat treatments (Figure 8F).

### High salinity suppresses *ELF3* alternative splicing

Salt stress influences plant growth and developmental processes, such as flowering time, which is closely associated with the clock function [51-53]. We therefore examined the effects of high salinity on the alternative splicing of the clock genes.

**Figure 9:**
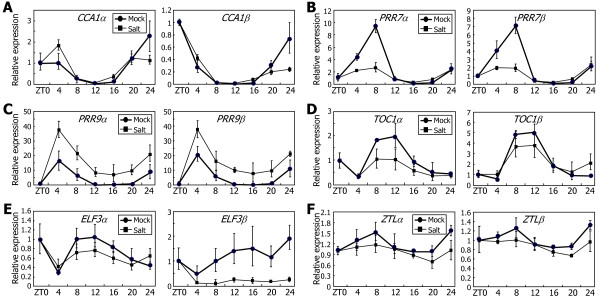
**Effects of high salinity on the alternative splicing of the clock genes.** Ten-day-old Col-0 plants grown on MS-agar plates under LDs were transferred to hydroponic MS medium containing 200 mM NaCl. The levels of the RNA splice variants of *CCA1***(A)**, *PRR7***(B)**, *PRR9***(C)**, *TOC1***(D)**, *ELF3***(E)**, and *ZTL***(F)** genes were determined by qRT-PCR. Biological triplicates were averaged. Bars indicate the standard error of the mean.

It appeared that *CCA1* and *ZTL* genes are not influenced by high salinity (Figure 9A and F, respectively). Notably, *PRR7* and *TOC1* genes were suppressed by high salinity. The levels of both *α* and *β* transcripts of these clock genes were reduced under high salt conditions (Figure 9B and D, respectively), showing that their alternative splicing is not influenced by high salinity. *ELF3* gene was also suppressed by high salinity (Figure 9E), but the reduction of *ELF3β* transcript level was more prominent than that of *ELF3α* level, showing that *ELF3* alternative splicing is suppressed by high salinity. The levels of *PRR9α* and *PRR9β* transcripts increased with high salinity (Figure 9C), indicating that *PRR9* gene is induced but its alternative splicing is not affected by high salinity.

## Discussion

### Effects of environmental conditions on the alternative splicing of the clock genes

Rhythmic expression of stress response genes and distinct phenotypes of clock mutants under abiotic stress conditions underscore the close connection between the circadian clock and environmental stress response in plants. One of the best-understood mechanisms is the clock control of C-*REPEAT BINDING FACTOR* (*CBF*) genes that play a pivotal role in cold stress response [18,54]. The central oscillators CCA1 and LHY regulate the expression of the *CBF* genes by binding directly to their gene promoters [55]. The *CBF* genes are also directly regulated by PRR9, PRR7, and PRR5 [56,57]. In addition, the transcription of CBF target genes, such as *COLD REGULATED 15 A* (*COR15A*) and *RESPONSIVE TO DISSECATION 29 A* (*RD29A*) [58,59], is clock-controlled [18].

Altered stress responses of various clock mutants further support the clock control of abiotic stress adaptation. The *prr9 prr7 prr5* triple mutants exhibit enhanced resistance to drought and cold stresses [60]. *TOC1*-deficient mutants display drought-tolerant phenotypes [61]. In addition, it has been shown that *Arabidopsis* plants that are defective in *CCA1*, *LHY*, and *GI* genes are susceptible to freezing temperatures [55,62].

Although the linkage between the clock and environmental stress responses has been widely explored, molecular mechanisms and underlying signaling schemes have not been studied at the molecular level in most cases. It has been reported that low temperatures reduce the amplitude of the clock gene expression [18]. Meanwhile, it is known that the clock genes are regulated by extensive alternative splicing, which is influenced by low temperatures. It is therefore evident that alternative splicing of the clock genes should be taken into the interpretation of the expression analysis data under abiotic stress conditions.

This study shows that a group of major clock genes undergoes alternative splicing through a variety of splicing modes, such as intron retention, exon skipping, and selection of alternative 5′ splice sites, resulting in two or more RNA splice variants for each clock gene. It was also found that photoperiod and abiotic stresses, such as temperature extremes and high salinity, broadly affect the alternative splicing of the clock genes. On the basis of the effects of CHX on the relative levels of RNA splice variants and expression assays in NMD-defective mutants, we propose that the alternative splicing of *CCA1*, *PRR7*, *PRR9*, and *ZTL* genes is productive with RNA splice variants encoding distinct proteins. In contrast, the RNA splice variants of *TOC1* and *ELF3* genes are predicted to be degraded through the NMD-mediated degradation pathway.

Collectively, our data strongly support the notion that the major clock genes are also regulated at the posttranscriptional level through alternative splicing in addition to the transcriptional control under both normal and environmental stress conditions. Alternative splicing-mediated control of the clock genes would serve as a molecular scheme that incorporates environmental stress signals into the clock, as has been verified with *CCA1* alternative splicing that links low temperature signals with the clock [27].

In this work, we focused on two major RNA splice variants of each clock gene, although additional RNA splice variants have been identified or predicted for some of the clock genes examined (Figure 1). More works on the additional RNA splice variants are required to further extend our understanding on the linkage between alternative splicing events of the clock genes and environmental stress responses. Searching for a full set of RNA splice variants of each clock gene, as has been performed by RNA sequencing method [20], will also be helpful for the elucidation of the clock function in abiotic stress adaptation. We are currently working on plants that are impaired in the alternative splicing of each clock gene and those expressing a specific RNA splice variant to investigate the physiological roles of the alternative splicing of the clock genes.

### Function of alternative protein isoforms

Recent studies have shown that protein isoforms that lack specific functional domains, which are produced by the alternative splicing of transcription factor genes, act as competitive inhibitors of the authentic transcription factors by forming nonfunctional heterodimers [27,63]. The best-characterized mechanism is the dominant negative regulation of the CCA1 transcription factor (CCA1α) by the protein isoform CCA1β. While the CCA1β isoform possesses protein domains required for dimmer formation and transcriptional activation, it lacks the MYB domain necessary for DNA binding [27]. Therefore, CCA1β is capable of interacting with CCA1α, forming CCA1α-CCA1β heterodimers that are excluded from DNA binding.

According to the domain structures of the protein isoforms encoded by the NMD-insensitive RNA splice variants, the PRR7β and PRR9β protein isoforms are predicted to function in a way that is distinct from that of CCA1β. Unlike CCA1β that lacks the MYB DNA-binding domain, PRR7β and PRR9β have the CCT domain, which is responsible for DNA binding, but lack the PR domain that mediates protein-protein interactions [7,23,45-47]. A plausible working mechanism of the PRR7β and PRR9β protein isoforms would be that they compete with the authentic PRR7α and PRR9α transcription factors for binding to the promoters of target genes, as has been previously proposed [64]. Further investigations are required to determine the functional modes of PRR7β and PRR9β.

Two RNA splice variants of *ZTL* gene are also insensitive to NMD, and two protein isoforms, ZTLα and ZTLβ, are expected to be produced. The ZTLα and ZTLβ isoforms are identical except for the far C-terminal sequences; the former is larger than the latter by 17 residues. The functional mode of ZTLβ thus might differ from the β protein isoforms of other clock components. The lack of the C-terminal extension might also influence the protein conformation of the ZTLβ isoform, which would affect its substrate specificity or enzymatic activity. The smaller ZTLβ isoform has been annotated as the authentic ZTL protein in the literature [23,65]. We found that the level of *ZTLβ* transcript is much higher than that of *ZTLα* transcript, which is in contrast to the *α*/*β* ratios of other clock genes. It is currently unclear whether ZTLα or ZTLβ or both is an authentic enzyme. Phenotypic and physiological assays on transgenic plants that specifically express either *ZTLα* or *ZTLβ* cDNA would help clarify this uncertainty.

### NMD-mediated control of the clock gene expression

Unlike the NMD-insensitive *β* transcripts of *CCA1*, *PRR7*, *PRR9*, and *ZTL* genes, *TOC1β* and *ELF3β* transcripts are apparently targeted by NMD. The *TOC1β* and *ELF3β* transcripts possess sequence features that are frequently observed in NMD substrates, in which they have splice junctions downstream of the PTC and very long 3′-UTRs [30].

Physiological roles of the NMD pathway are somewhat controversial. According to the “noise” hypothesis, NMD-sensitive RNA splice variants occur as a result of splicing error and are eventually removed through the NMD pathway [66]. In contrast, in the “regulated unproductive splicing and translation (RUST)” hypothesis, alternative splicing is coupled with NMD as a regulatory mechanism for monitoring the abundance of full-size RNA splice variants [67]. We believe that the RUST hypothesis fits well into the alternative splicing of the *TOC1* and *ELF3* genes, based on the following reasons. First, the RUST hypothesis depicts that alternative splicing occurs through distinct modes of splicing events [26,34]. We found that the alternative splicing of the *TOC1* and *ELF3* genes is mediated by the retention of specific introns, supporting the notion that their alternative splicing is a regulated process rather than a simple splicing error. Second, their alternative splicing is regulated by environmental factors in a discrete manner. Production of the *ELF3β* transcript is suppressed by cold and high salinity conditions but induced under heat stress conditions. Third, whereas their alternative splicing is markedly influenced by abiotic stresses, the levels of *TOC1α* and *ELF3α* transcripts are less affected under identical conditions. However, it is still possible that the RNA splice variants may be at least in part translated into proteins. It has recently been reported that some NMD targets are stabilized and translated into proteins under certain conditions [68].

## Conclusions

We investigated the alternative splicing events of major clock genes under various environmental conditions and the sensitivity of their RNA splice variants to NMD. Alternative splicing patterns of the clock genes were differently affected by changes in photoperiod and abiotic stresses, such as cold, heat, and high salinity. Based on the results of this study, we propose that alternative splicing of the clock genes, either by producing truncated isoforms that act as self-regulators or by regulating the abundance of full-size transcripts at the posttranscriptional level, contributes to the precise regulation of the clock function, particularly under fluctuating environmental conditions. It may also serve as a web that integrates environmental stress signals into the clock, providing an adaptation strategy in response to unpredictable environmental changes.

## Methods

### Bioinformatics software

Gene sequences and their exon-intron structures were obtained from the *Arabidopsis* Information Resource (TAIR, http://www.arabidopsis.org/). Alternative splicing modes of *PRR7* and *TOC1* genes, which have not been annotated in TAIR, were predicted based on the sequence analysis and the previous reports describing the predicted types of alternative splicing [26,34]. For *ELF3* gene that has not been studied, the presence of alternatively spliced RNA variants was verified by direct sequencing of RT-PCR products. Protein domain structures were predicted using the SMART (http://smart.embl-heidelberg.de/) and Pfam (http://www.sanger.ac.uk/Software/Pfam/) databases.

### Plant materials and growth conditions

*Arabidopsis thaliana* ecotype Columbia-0 (Col-0) was used, unless otherwise specified. The *upf1*-*5* and *upf3*-*1* mutants, which have been previously described [26,34], were kindly provided by Dr. Jeong Sheop Shin (Korea University, Seoul, Korea) and Dr. Hee-Jeong Jeong (Kyung Hee University, Yongin, Korea). Plants were grown on ½ X Murashige & Skoog media containing 0.6% (w/v) agar (hereafter referred to as MS-agar plates) in a growth chamber set at 23°C with relative humidity of 60% under either long day conditions (LDs, 16-h light and 8-h dark) or short day conditions (SDs, 8-h light and 16-h dark) with white light illumination (120 μM photons m^-2^ s^-1^) provided by fluorescent FLR40D/A tubes (Osram, Seoul, Korea).

### Analysis of gene transcript levels

Extraction of total RNA samples from appropriate plant materials and RT-PCR conditions have been described previously [69]. Total RNA samples were extensively pretreated with an RNase-free DNase to eliminate contaminating genomic DNA prior to analysis.

Quantitative real-time RT-PCR (qRT-PCR) was employed to determine the levels of gene transcripts. RNA sample preparation, reverse transcription, and quantitative PCR were conducted according to the rules that have been proposed to ensure reproducible and accurate measurements [70].

qRT-PCR reactions were performed in 96-well blocks using an Applied Biosystems 7500 Real-Time PCR System (Foster City, CA) using the SYBR Green I master mix in a volume of 20 μl. The PCR primers were designed using the Primer Express Software installed in the system and listed in Additional file 8. The two-step thermal cycling profile used was 15 s at 94°C and 1 min at 68°C. The *eIF4A* gene (At3g13920) was included in the reactions as internal control to normalize the variations in the amounts of cDNA used [71]. All qRT-PCR reactions were performed in biological triplicates using RNA samples extracted from three independent plant materials grown under identical conditions. The comparative ΔΔC_T_ method was used to evaluate the relative quantities of each amplified product in the samples. The threshold cycle (C_T_) was automatically determined for each reaction using the default parameters of the system. The specificity of PCR reactions was determined by melt curve analysis of the amplified products using the standard methods installed in the system.

### Absolute quantification of gene transcripts

Absolute quantification of gene transcripts was conducted as previously described [27]. The cDNAs of alternatively spliced RNA variants were subcloned into a pGADT7 vector (Clontech, Mountain View, CA), and the absolute standard curve of each transcript was obtained by a series of 10-fold dilutions covering from 10^-19^ to 10^-23^ mol/μl, as described elsewhere [36,37]. Quantitative RT-PCR was conducted using a SYBR Green I master mix (Applied Biosystems) with splice variant-specific primers listed in Additional file 8.

### Abiotic stress treatments

*Arabidopsis* plants grown for 10 days on MS-agar plates under LDs were used for abiotic stress treatments. For cold and heat treatments, plants were incubated at 4°C or at 37°C under continuous light conditions for appropriate time durations before harvesting plant materials. To examine the effects of high salinity on the alternative splicing of the clock genes, plants were transferred to MS liquid medium containing 200 mM NaCl under continuous light conditions for appropriate time durations.

### Cycloheximide (CHX) treatments

The CHX treatments were performed as described elsewhere [29,30]. Ten-day-old plants grown on MS-agar plates were transferred to MS liquid medium containing 20 μM CHX. Following vacuum infiltration for 10 min, the plants were incubated for 5 h at 23°C under normal growth conditions before harvesting plant materials for total RNA extraction.

## Competing interests

The authors declare that they have no competing interests.

## Authors’ contributions

CMP and YJK conceptualized the project and analyzed the data. CMP, YJK, and MJP wrote the manuscript. YJK and MJP carried out the molecular assays on the alternative splicing of the clock genes. YJK, SGK, and ITB predicted the alternative splicing patterns of the clock genes. All authors discussed the results and approved the final form of the manuscript.

## Supplementary Material

Additional file 1 **Nucleotide sequence comparison of *PRR7 *** **gDNA and *PRR7β *** **cDNA.** The nucleotide sequence of *PRR7β* cDNA was determined by DNA sequencing of RT-PCR product and aligned with *PRR7* genomic DNA (*PRR7* gDNA) using the ClustalW software (http://www.ebi.ac.uk/tools/msa/clustalw2/). Part of the aligned sequences containing exons 1, 2, 3, and 4 and introns 2, 3, and 4 was displayed. Intron 3, which is retained in the *PRR7β* transcript as a result of alternative splicing, is underlined (blue). A PTC (premature termination codon) is introduced into the *PRR7β* transcript (red asterisk). Click here for file

Additional file 2 **Nucleotide sequence comparison of *PRR9 *** **gDNA and *PRR9β *** **cDNA.** The nucleotide sequence of *PRR9β* cDNA was determined by DNA sequencing of RT-PCR product and aligned with *PRR9* gDNA using the ClustalW software. Part of the aligned sequences containing exons 1, 2, 3, 4, and 5 and introns 1, 2, 3, and 4 was displayed. The alternative splice site, which is used to produce the *PRR9β* transcript, is underlined. Sequence analysis revealed that the *PRR9β* transcript occurs by the alternative 5′ splice site in intron 2 (blue). Click here for file

Additional file 3 **Nucleotide sequence comparison of *TOC1 *** **gDNA and *TOC1β *** **cDNA.** The nucleotide sequence of *TOC1β* cDNA was determined by DNA sequencing of RT-PCR product and aligned with *TOC1* gDNA using the ClustalW software. Part of the aligned sequences containing exons 4, 5, and 6 and introns 3, 4, and 5 was displayed. The retained intron 4, which is included in the *TOC1β* transcript as a result of alternative splicing, is underlined (blue). A PTC is introduced into the *TOC1β* transcript (red asterisk). Click here for file

Additional file 4 **Nucleotide sequence comparison of *ELF3 *** **gDNA and *ELF3β *** **cDNA.** The nucleotide sequence of *ELF3β* cDNA was determined by DNA sequencing of RT-PCR product and aligned with *ELF3* gDNA using the ClustalW software. Part of the aligned sequences containing exons 2 and 3 and intron 2 was displayed. The alternative exon within intron 2, which is included in the *ELF3β* transcript as a result of alternative splicing, is underlined. Red boxes indicate conserved ‘T’ and 'AG’ sequences at the 5′ and 3′ ends of introns. Sequence analysis revealed that the *ELF3β* transcript occurs by the inclusion of an alternative exon consisting of 178 nucleotides within intron 2 (blue). A PTC is introduced into the *ELF3β* transcript (red asterisk). Click here for file

Additional file 5 **Nucleotide sequence comparison of *ZTL *** **gDNA and *ZTLα *** **and *ZTLβ *** **cDNAs.** The nucleotide sequences of *ZTLα* and *ZTLβ* cDNAs were determined by DNA sequencing of RT-PCR products and aligned with *ZTL* gDNA using the ClustalW software. Part of the aligned sequences containing exons 2 and 3 and intron 2 was displayed. Intron 2, which is retained in the *ZTLβ* transcript as a result of alternative splicing, is underlined (blue). A PTC is introduced into the *ZTLβ* transcript (red asterisk). The 3′ untranslated region of the *ZTLβ* transcript is shown in gray. Click here for file

Additional file 6 **The fate of *TOC1β *** **and *ELF3β *** **transcripts under heat stress conditions.** Ten-day-old Col-0 plants and *upf1-5* and *upf3-1* mutants grown on ½ X Murashige & Skoog media containing 0.6% (w/v) agar plates (hereafter referred to as MS-agar plates) were transferred to 37°C for 12 h before harvesting whole plant materials for the extraction of total RNA. Levels of *TOC1β* and *ELF3β* transcripts were determined by quantitative real-time RT-PCR (qRT-PCR). Biological triplicates were averaged and statistically treated using Student *t*-test (*P<0.01). Bars indicate standard error of the mean. Click here for file

Additional file 7 **The fate of *ZTLα *** **transcript.** Plants were grown on MS-agar plates for 10 days under normal growth conditions. Ten-day-old Col-0 plants were transferred to liquid MS culture containing 20 μM cycloheximide (CHX). Following vacuum infiltration for 10 min, the plants were incubated for 5 h at 23°C under normal growth conditions before harvesting whole plant materials for the extraction of total RNA (left panel). The *upf1*-*5* and *upf3*-*1* mutants were not treated with CHX (right panel). Levels of *ZTLα* transcript were determined by qRT-PCR Biological triplicates were averaged. Bars indicate standard error of the mean. Click here for file

Additional file 8 **Primers used in qRT-PCR, RT-PCR, and gene cloning.** F, forward primer; R, reverse primer. Click here for file
