# Supplementary material for: Alternative splicing and nonsense-mediated decay of circadian clock genes under environmental stress conditions in Arabidopsis
Source: BMC Plant Biol. 2014 May 19;14:136. doi: 10.1186/1471-2229-14-136 (PMC4035800; doi:10.1186/1471-2229-14-136)
Supplement: Additional file 1 — Nucleotide sequence comparison of PRR7 gDNA and PRR7β cDNA. The nucleotide sequence of PRR7β cDNA was determined by DNA sequencing of RT-PCR product and aligned with PRR7 genomic DNA (PRR7 gDNA) using the ClustalW software (http://www.ebi.ac.uk/tools/msa/clustalw2/). Part of the aligned sequences containing exons 1, 2, 3, and 4 and introns 2, 3, and 4 was displayed. Intron 3, which is retained in the PRR7β transcript as a result of alternative splicing, is underlined (blue). A PTC (premature termination codon) is introduced into the PRR7β transcript (red asterisk). [file 1471-2229-14-136-S1.pdf]

Additional file 1

```
PRR7 gDNA    ATGAATGCTAATGAGGAGGGGGAGGGTTCACGTTACCCAATCACTGATCGAAAGACCGGAGAGACGAAATTCGATAGGGTTGAGAGTCGGACAGAGAAGCATAGTGAAGAAGAGAAAACT 120
PRR7β        ATGAATGCTAATGAGGAGGGGGAGGGTTCACGTTACCCAATCACTGATCGAAAGACCGGAGAGACGAAATTCGATAGGGTTGAGAGTCGGACAGAGAAGCATAGTGAAGAAGAGAAAACT 120

PRR7 gDNA    AATGGAATTACTATGGATGTGAGAAATGGGAGTTCAGGTGGACTGCAAATTCCATTGTCGCAACAAACAGCGGCAACTGTCTGTTGGGAAAGGTTTCTTCATGTGAGAACCATTAGAGTT 240
PRR7β        AATGGAATTACTATGGATGTGAGAAATGGGAGTTCAGGTGGACTGCAAATTCCATTGTCGCAACAAACAGCGGCAACTGTCTGTTGGGAAAGGTTTCTTCATGTGAGAACCATTAGAGTT 240

                                                                  Exon 1
PRR7 gDNA    CTGCTTGTCGAAAATGACGACTGCACTCGTTATATCGTTACTGCACTTCTTCGCAATTGTAGCTATGAAGGTCAGTTTTGAAGCCTATGGCCCAACTTTAATCTATAGCGCATATATGTA 360
PRR7β        CTGCTTGTCGAAAATGACGACTGCACTCGTTATATCGTTACTGCACTTCTTCGCAATTGTAGCTATGAAG-------------------------------------------------- 310

                                  Intron 2
PRR7 gDNA    CCCGTTTCGGTTCTGTTTGTTGATTGATTATTATTAACTCTGTCATGGCAGTTGTTGAGGCGTCAAATGGGATACAAGCTTGGAAGGTGTTAGAAGATCTAAACAATCATATTGATATTG 480
PRR7β        ---------------------------------------------------TTGTTGAGGCGTCAAATGGGATACAAGCTTGGAAGGTGTTAGAAGATCTAAACAATCATATTGATATTG 379

                                                                                   Exon 2
PRR7 gDNA    TGCTAACAGAGGTGATCATGCCTTACTTATCTGGTATCGGTCTCTTGTGCAAGATTTTGAACCACAAATCTCGTCGGAACATCCCTGTCATCAGTGAGTTCTTTTTCCTTGGTCGTTTTA 600
PRR7β        TGCTAACAGAGGTGATCATGCCTTACTTATCTGGTATCGGTCTCTTGTGCAAGATTTTGAACCACAAATCTCGTCGGAACATCCCTGTCATCAGTGAGTTCTTTTTCCTTGGTCGTTTTA 499

PRR7 gDNA    CATTGAGCTCTTTCTTTTGAAGTTACACGATTTGTTGAGTCTTCTCTAGCGTATGTTGGAAAGTAGATGCTTTTAACTACATTCCCCTGTGAGATTTGTGTTGCAGTGATGTCATCTCAT 720
PRR7β        CATTGAGCTCTTTCTTTTGAAGTTACACGATTTGTTGAGTCTTCTCTAGCGTATGTTGGAAAGTAGATGCTTTTAACTACATTCCCCTGTGAGATTTGTGTTGCAGTGATGTCATCTCAT 619
                               *                    Retention of intron 3
                                                                                                    Exon 3
PRR7 gDNA    GACTCAATGGGGCTGGTCTTTAAGTGCTTATCGAAAGGAGCTGTTGACTTTCTTGTTAAGCCAATAAGAAAAAATGAGCTTAAGATCCTTTGGCAGCATGTTTGGAGAAGATGCCAAAGT 840
PRR7β        GACTCAATGGGGCTGGTCTTTAAGTGCTTATCGAAAGGAGCTGTTGACTTTCTTGTTAAGCCAATAAGAAAAAATGAGCTTAAGATCCTTTGGCAGCATGTTTGGAGAAGATGCCAAAGT 739

PRR7 gDNA    GTATGTCCTTGCTCATATATGATTAATCTGAAAACCTGTTGGTACAACTGGTGATAAGTAGTAAACTAGAAATTCATGGTCTAATTGTGGATTGGTATCTTCTTTTTTTTTTTCCACATG 960
PRR7β        ------------------------------------------------------------------------------------------------------------------------

                      Intron 4
PRR7 gDNA    ATTGGTATCTTACTTTTTGGTTTCTCATGGTTTTCCTTGTTTCTTGTTCAGTCTAGTGGTAGTGGAAGTGAGAGCGGAACGCATCAAACTCAAAAGTCTGTGAAATCGAAAAGTATTAAA 1080
PRR7β        ---------------------------------------------------TCTAGTGGTAGTGGAAGTGAGAGCGGAACGCATCAAACTCAAAAGTCTGTGAAATCGAAAAGTATTAAA 808

                                                                                 Exon 4
PRR7 gDNA    AAATCTGATCAAGATTCAGGAAGCAGTGATGAGAATGAAAATGGGAGCATTGGCCTGAATGCTAGTGATGGAAGTAGTGATGGGAGTGGCGCTCAG 1176
PRR7β        AAATCTGATCAAGATTCAGGAAGCAGTGATGAGAATGAAAATGGGAGCATTGGCCTGAATGCTAGTGATGGAAGTAGTGATGGGAGTGGCGCTCAG 904
```

Additional file 1. Nucleotide sequence comparison of *PRR7* gDNA and *PRR7β* cDNA.
The nucleotide sequence of *PRR7β* cDNA was determined by DNA sequencing of RT-PCR product and aligned with *PRR7* genomic DNA (*PRR7* gDNA) using the ClustalW software (http://www.ebi.ac.uk/tools/msa/clustalw2/). Part of the aligned sequences containing exons 1, 2, 3, and 4 and introns 2, 3, and 4 was displayed. Intron 3, which is retained in the *PRR7β* transcript as a result of alternative splicing, is underlined (blue). A PTC (premature termination codon) is introduced into the *PRR7β* transcript (red asterisk).
