# Supplementary material for: Alternative splicing and nonsense-mediated decay of circadian clock genes under environmental stress conditions in Arabidopsis
Source: BMC Plant Biol. 2014 May 19;14:136. doi: 10.1186/1471-2229-14-136 (PMC4035800; doi:10.1186/1471-2229-14-136)
Supplement: Additional file 2 — Nucleotide sequence comparison of PRR9 gDNA and PRR9β cDNA. The nucleotide sequence of PRR9β cDNA was determined by DNA sequencing of RT-PCR product and aligned with PRR9 gDNA using the ClustalW software. Part of the aligned sequences containing exons 1, 2, 3, 4, and 5 and introns 1, 2, 3, and 4 was displayed. The alternative splice site, which is used to produce the PRR9β transcript, is underlined. Sequence analysis revealed that the PRR9β transcript occurs by the alternative 5′ splice site in intron 2 (blue). [file 1471-2229-14-136-S2.pdf]

Additional file 2

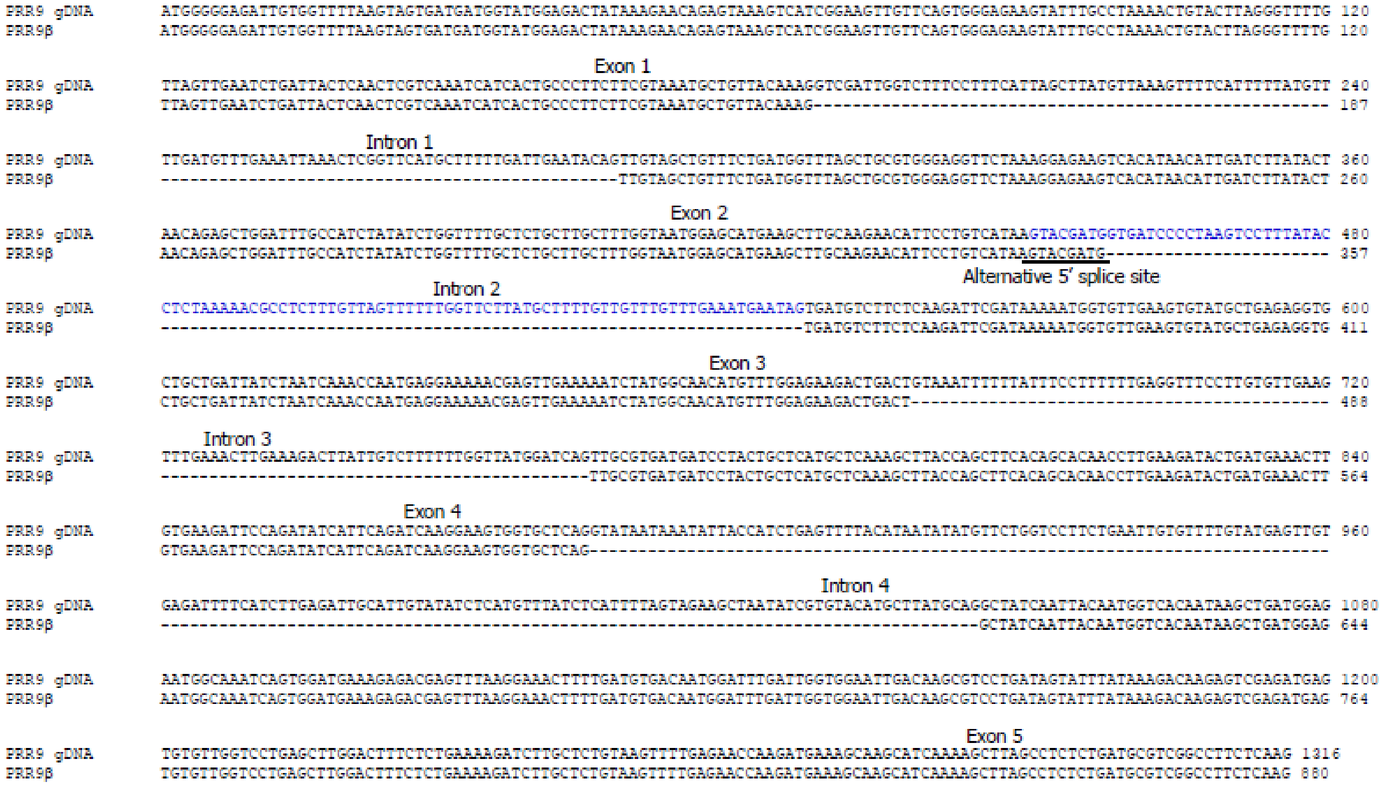

Additional file 2. Nucleotide sequence comparison of *PRR9* gDNA and *PRR9β* cDNA.
The nucleotide sequence of *PRR9β* cDNA was determined by DNA sequencing of RT-PCR product and aligned with *PRR9* gDNA using the ClustalW software. Part of the aligned sequences containing exons 1, 2, 3, 4, and 5 and introns 1, 2, 3, and 4 was displayed. The alternative splice site, which is used to produce the *PRR9β* transcript, is underlined. Sequence analysis revealed that the *PRR9β* transcript occurs by the alternative 5' splice site in intron 2 (blue).
