# Supplementary material for: Alternative splicing and nonsense-mediated decay of circadian clock genes under environmental stress conditions in Arabidopsis
Source: BMC Plant Biol. 2014 May 19;14:136. doi: 10.1186/1471-2229-14-136 (PMC4035800; doi:10.1186/1471-2229-14-136)
Supplement: Additional file 3 — Nucleotide sequence comparison of TOC1 gDNA and TOC1β cDNA. The nucleotide sequence of TOC1β cDNA was determined by DNA sequencing of RT-PCR product and aligned with TOC1 gDNA using the ClustalW software. Part of the aligned sequences containing exons 4, 5, and 6 and introns 3, 4, and 5 was displayed. The retained intron 4, which is included in the TOC1β transcript as a result of alternative splicing, is underlined (blue). A PTC is introduced into the TOC1β transcript (red asterisk). [file 1471-2229-14-136-S3.pdf]

Additional file 3

```
TOC1 gDNA      GTTTGTTTTGCTTGTCTCAAGTTCTCTCTATTTATCTTACTGTTTTTGAGCAATACTTGATAGCCATATCTCTCAATCTGTCGTCTTCTGAAATAAAAGACCTTGATGCATGAAGTTAAT 120
TOC1β          ------------------------------------------------------------------------------------------------------------------------

                        Intron 3
TOC1 gDNA      GCTATTGATTTCATATATTTGTTTTGGCAGCTAGGACTTGCTGAGAAGAATATGTTGAGCTATGATTTTGATCTTGTGGGATCTGATCAAAGTGATCCAAACACAAATAGTACCAACCTG 240
TOC1β          ------------------------------CTAGGACTTGCTGAGAAGAATATGTTGAGCTATGATTTTGATCTTGTGGGATCTGATCAAAGTGATCCAAACACAAATAGTACCAACCTG 90

                                                                           Exon 4
TOC1 gDNA      TTCTCTGACGACACAGATGATAGAAGTCTTAGGTCCACCAACCCACAGAGAGGAAATTTAAGTCACCAGGAAAATGAGGTGAGAAAGTATTTGACAGTTTAAAGCCCTCTGAAAATGGAG 360
TOC1β          TTCTCTGACGACACAGATGATAGAAGTCTTAGGTCCACCAACCCACAGAGAGGAAATTTAAGTCACCAGGAAAATGAGGTGAGAAAGTATTTGACAGTTTAAAGCCCTCTGAAAATGGAG 210
                                                                                                          *

TOC1 gDNA      AAGAACTGATTTTTTAGCTTGCGCTCATGCTTTTGCTGGTAGTGGTCTGTTGCTACTGCTCCTGTTCATGCTCGTGATGGTGGTCTTGGTGCTGATGGAACAGCCACTTCTTCTCTTGCT 480
TOC1β          AAGAACTGATTTTTTAGCTTGCGCTCATGCTTTTGCTGGTAGTGGTCTGTTGCTACTGCTCCTGTTCATGCTCGTGATGGTGGTCTTGGTGCTGATGGAACAGCCACTTCTTCTCTTGCT 330
                      Retention of intron 4
                                                                    Exon 5
TOC1 gDNA      GTTACTGCTATAGAGCCTCCATTGGATCATCTTGCTGGGTCTCACCATGAGCCAATGAAAAGAAATAGTAATCCAGGTGATTGTTTCTCTTTGATGTCTCACTTTCTAATAGCTAGTCTC 600
TOC1β          GTTACTGCTATAGAGCCTCCATTGGATCATCTTGCTGGGTCTCACCATGAGCCAATGAAAAGAAATAGTAATCCAG-------------------------------------------- 406

                                         Intron 5
TOC1 gDNA      TAAAAGAACCTTTGTTTTAGTGAATTCTAATATAGTAGGGGTTTTGCAGCGCAATTTTCTTCAGCACCGAAGAAAAGTAGATTGAAGATCGGAGAGTCCTCTGCTTTCTTTACATATGTC 720
TOC1β          -------------------------------------------------CGCAATTTTCTTCAGCACCGAAGAAAAGTAGATTGAAGATCGGAGAGTCCTCTGCTTTCTTTACATATGTC 477

TOC1 gDNA      AAATCTACTGTCCTTAGAACTAACGGTCAGGATCCTCCTCTTGTCGATGGAAATGGCTCACTTCATCTTCATCGGGGTTTGGCGGAGAAGTTTCAAGTGGTGGCTAGTGAAGGGATCAAC 840
TOC1β          AAATCTACTGTCCTTAGAACTAACGGTCAGGATCCTCCTCTTGTCGATGGAAATGGCTCACTTCATCTTCATCGGGGTTTGGCGGAGAAGTTTCAAGTGGTGGCTAGTGAAGGGATCAAC 597

TOC1 gDNA      AACACCAAACAAGCACGCAGAGCAACACCAAAATCTACTGTCCTTAGAACTAACGGTCAGGATCCTCCTCTTGTCAATGGAAATGGCTCACATCATCTTCATCGGGGTGCGGCGGAAAAG 960
TOC1β          AACACCAAACAAGCACGCAGAGCAACACCAAAATCTACTGTCCTTAGAACTAACGGTCAGGATCCTCCTCTTGTCAATGGAAATGGCTCACATCATCTTCATCGGGGTGCGGCGGAAAAG 717

                                                                                  Exon 6
TOC1 gDNA      TTTCAAGTGGTGGCTAGTGAAGGGATCAACAACACCAAACAAGCACACAGAAGTAGAGGGACCGAGCAATACCATTCTCAAGGAGAGACCTTGCAGAATGGCGCCAGCTATCCACATTCC 1080
TOC1β          TTTCAAGTGGTGGCTAGTGAAGGGATCAACAACACCAAACAAGCACACAGAAGTAGAGGGACCGAGCAATACCATTCTCAAGGAGAGACCTTGCAGAATGGCGCCAGCTATCCACATTCC 837

TOC1 gDNA      CTTGAGCGGTCACGCACGCTTCCCACATCAATGGAATCTCATGGTAGGAACTACCAAGAGGGCAATATGAATATTCCCCAAGTTGCTATGAACAGAAGTAAAGATTCGTCTCAAGTTGAT 1200
TOC1β          CTTGAGCGGTCACGCACGCTTCCCACATCAATGGAATCTCATGGTAGGAACTACCAAGAGGGCAATATGAATATTCCCCAAGTTGCTATGAACAGAAGTAAAGATTCGTCTCAAGTTGAT 957

TOC1 gDNA      GGATCGGGTTTCTCTGCACCAAATGCCTATCCTTACTATATGCATGGGGTCATGAACCAAGTTATGATGCAATCAGCAGCCATGATGCCTCAATATGGTCATCAAATTCCTCATTGCCAA 1320
TOC1β          GGATCGGGTTTCTCTGCACCAAATGCCTATCCTTACTATATGCATGGGGTCATGAACCAAGTTATGATGCAATCAGCAGCCATGATGCCTCAATATGGTCATCAAATTCCTCATTGCCAA 1077

TOC1 gDNA      CCAAATCATCCGAATGGAATGACGGGATATCCTTACTACCACCACCCAATGAACACATCTTTGCAGCATAGTCAGATGTCTTTACAGAATGGTCAGATGTCTATGGTTCATCATTCTTGG 1440
TOC1β          CCAAATCATCCGAATGGAATGACGGGATATCCTTACTACCACCACCCAATGAACACATCTTTGCAGCATAGTCAGATGTCTTTACAGAATGGTCAGATGTCTATGGTTCATCATTCTTGG 1197
```

Additional file 3. Nucleotide sequence comparison of *TOC1* gDNA and *TOC1β* cDNA.

The nucleotide sequence of *TOC1β* cDNA was determined by DNA sequencing of RT-PCR product and aligned with *TOC1* gDNA using the ClustalW software. Part of the aligned sequences containing exons 4, 5, and 6 and introns 3, 4, and 5 was displayed. The retained intron 4, which is included in the *TOC1β* transcript as a result of alternative splicing, is underlined (blue). A PTC is introduced into the *TOC1β* transcript (red asterisk).
