# Supplementary material for: Alternative splicing and nonsense-mediated decay of circadian clock genes under environmental stress conditions in Arabidopsis
Source: BMC Plant Biol. 2014 May 19;14:136. doi: 10.1186/1471-2229-14-136 (PMC4035800; doi:10.1186/1471-2229-14-136)
Supplement: Additional file 4 — Nucleotide sequence comparison of ELF3 gDNA and ELF3β cDNA. The nucleotide sequence of ELF3β cDNA was determined by DNA sequencing of RT-PCR product and aligned with ELF3 gDNA using the ClustalW software. Part of the aligned sequences containing exons 2 and 3 and intron 2 was displayed. The alternative exon within intron 2, which is included in the ELF3β transcript as a result of alternative splicing, is underlined. Red boxes indicate conserved ‘T’ and 'AG’ sequences at the 5′ and 3′ ends of introns. Sequence analysis revealed that the ELF3β transcript occurs by the inclusion of an alternative exon consisting of 178 nucleotides within intron 2 (blue). A PTC is introduced into the ELF3β transcript (red asterisk). [file 1471-2229-14-136-S4.pdf]

Additional file 4

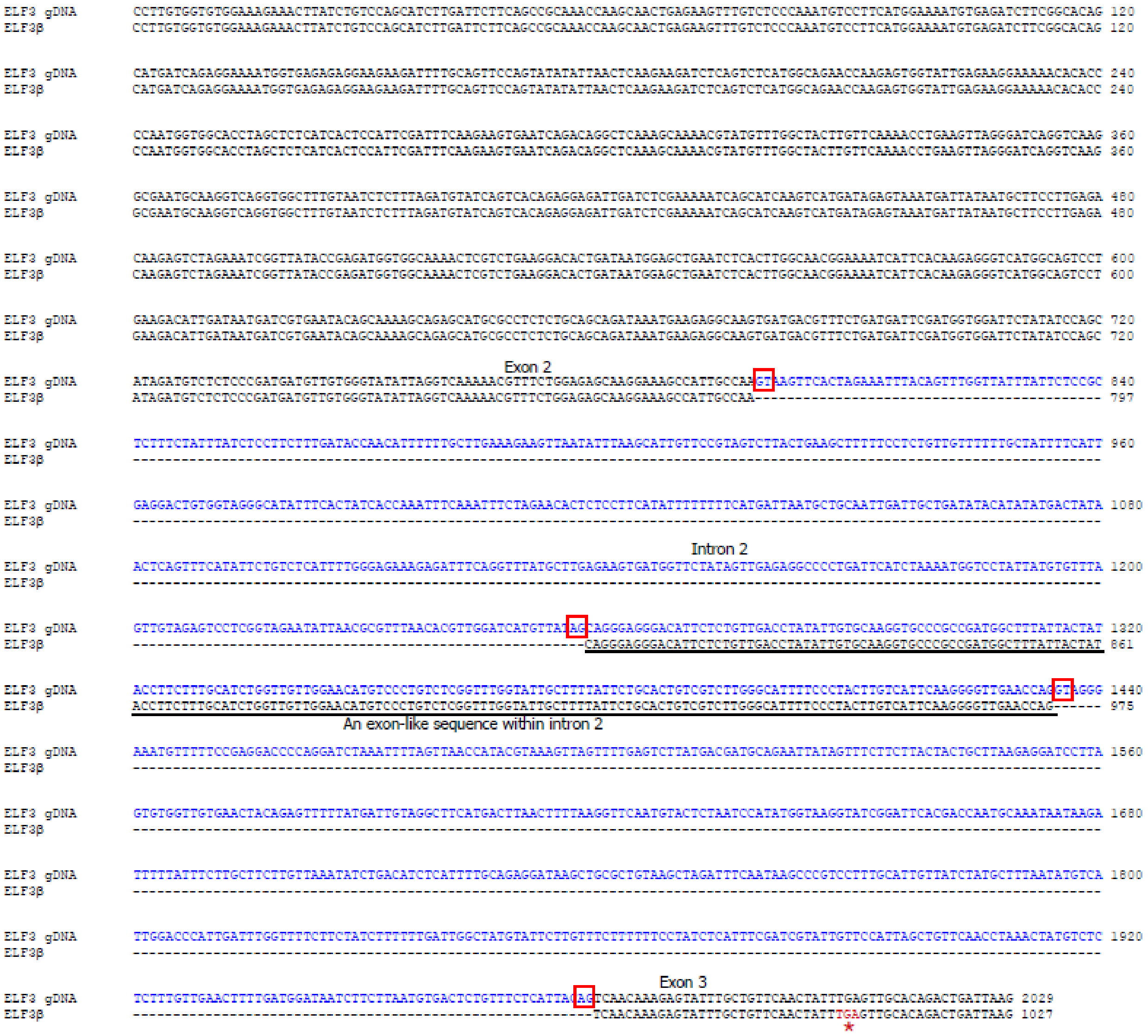

Additional file 4. Nucleotide sequence comparison of *ELF3* gDNA and *ELF3β* cDNA.
The nucleotide sequence of *ELF3β* cDNA was determined by DNA sequencing of RT-PCR product and aligned with *ELF3* gDNA using the ClustalW software. Part of the aligned sequences containing exons 2 and 3 and intron 2 was displayed. The alternative exon within intron 2, which is included in the *ELF3β* transcript as a result of alternative splicing, is underlined. Red boxes indicate conserved 'GT' and 'AG' sequences at the 5' and 3' ends of introns. Sequence analysis revealed that the *ELF3β* transcript occurs by the inclusion of an alternative exon consisting of 178 nucleotides within intron 2 (blue). A PTC is introduced into the *ELF3β* transcript (red asterisk).
