# Supplementary material for: Alternative splicing and nonsense-mediated decay of circadian clock genes under environmental stress conditions in Arabidopsis
Source: BMC Plant Biol. 2014 May 19;14:136. doi: 10.1186/1471-2229-14-136 (PMC4035800; doi:10.1186/1471-2229-14-136)
Supplement: Additional file 5 — Nucleotide sequence comparison of ZTL gDNA and ZTLα and ZTLβ cDNAs. The nucleotide sequences of ZTLα and ZTLβ cDNAs were determined by DNA sequencing of RT-PCR products and aligned with ZTL gDNA using the ClustalW software. Part of the aligned sequences containing exons 2 and 3 and intron 2 was displayed. Intron 2, which is retained in the ZTLβ transcript as a result of alternative splicing, is underlined (blue). A PTC is introduced into the ZTLβ transcript (red asterisk). The 3′ untranslated region of the ZTLβ transcript is shown in gray. [file 1471-2229-14-136-S5.pdf]

Additional file 5

```
ZTL gDNA    TTGGCGAAGGGTTTGTCAGAATGCATGGGGAAGTGAAACAACACGGGTATTAGAGACTGTTCCTGGTGCTAAAAGACTCGGTTGGGGCCGCTTGGCTAGAGAACTGACCACCCTTGAAGC 120
ZTLα        TTGGCGAAGGGTTTGTCAGAATGCATGGGGAAGTGAAACAACACGGGTATTAGAGACTGTTCCTGGTGCTAAAAGACTCGGTTGGGGCCGCTTGGCTAGAGAACTGACCACCCTTGAAGC 120
ZTLβ        TTGGCGAAGGGTTTGTCAGAATGCATGGGGAAGTGAAACAACACGGGTATTAGAGACTGTTCCTGGTGCTAAAAGACTCGGTTGGGGCCGCTTGGCTAGAGAACTGACCACCCTTGAAGC 120

ZTL gDNA    TGCAGCTTGGAGAAAACTTTCGGTTGGAGGTTCTGTTGAGCCTTCTCGCTGTAATTTTAGTGCTTGTGCTGTCGGGAACAGAGTTGTTCTGTTTGGAGGAGAAGGTGTGAATATGCAGCC 240
ZTLα        TGCAGCTTGGAGAAAACTTTCGGTTGGAGGTTCTGTTGAGCCTTCTCGCTGTAATTTTAGTGCTTGTGCTGTCGGGAACAGAGTTGTTCTGTTTGGAGGAGAAGGTGTGAATATGCAGCC 240
ZTLβ        TGCAGCTTGGAGAAAACTTTCGGTTGGAGGTTCTGTTGAGCCTTCTCGCTGTAATTTTAGTGCTTGTGCTGTCGGGAACAGAGTTGTTCTGTTTGGAGGAGAAGGTGTGAATATGCAGCC 240

ZTL gDNA    TATGAATGATACCTTTGTTTTAGATCTGAATTCTGATTACCCTGAATGGCAACATGTCAAAGTGAGCTCCCCTCCTCCAGGACGCTGGGGCCACACGCTCACTTGTGTAAATGGCTCCAA 360
ZTLα        TATGAATGATACCTTTGTTTTAGATCTGAATTCTGATTACCCTGAATGGCAACATGTCAAAGTGAGCTCCCCTCCTCCAGGACGCTGGGGCCACACGCTCACTTGTGTAAATGGCTCCAA 360
ZTLβ        TATGAATGATACCTTTGTTTTAGATCTGAATTCTGATTACCCTGAATGGCAACATGTCAAAGTGAGCTCCCCTCCTCCAGGACGCTGGGGCCACACGCTCACTTGTGTAAATGGCTCCAA 360

ZTL gDNA    TCTGGTTGTATTTGGTGGCTGTGGTCAGCAAGGTTTGCTGAACGATGTGTTTGTCTTGAATCTCGATGCTAAGCCGCCTACTTGGAGGGAAATTTCGGGTTTGGCTCCGCCTCTACCAAG 480
ZTLα        TCTGGTTGTATTTGGTGGCTGTGGTCAGCAAGGTTTGCTGAACGATGTGTTTGTCTTGAATCTCGATGCTAAGCCGCCTACTTGGAGGGAAATTTCGGGTTTGGCTCCGCCTCTACCAAG 480
ZTLβ        TCTGGTTGTATTTGGTGGCTGTGGTCAGCAAGGTTTGCTGAACGATGTGTTTGTCTTGAATCTCGATGCTAAGCCGCCTACTTGGAGGGAAATTTCGGGTTTGGCTCCGCCTCTACCAAG 480

ZTL gDNA    ATCATGGCACAGCTCATGCACCCTTGACGGAACCAAACTGATAGTCTCTGGAGGCTGTGCAGATTCCGGCGTTCTTCTAAGTGACACGTTCCTCCTCGACCTTTCAATAGAGAAACCAGT 600
ZTLα        ATCATGGCACAGCTCATGCACCCTTGACGGAACCAAACTGATAGTCTCTGGAGGCTGTGCAGATTCCGGCGTTCTTCTAAGTGACACGTTCCTCCTCGACCTTTCAATAGAGAAACCAGT 600
ZTLβ        ATCATGGCACAGCTCATGCACCCTTGACGGAACCAAACTGATAGTCTCTGGAGGCTGTGCAGATTCCGGCGTTCTTCTAAGTGACACGTTCCTCCTCGACCTTTCAATAGAGAAACCAGT 600

                                                                      Exon 2
ZTL gDNA    GTGGAGAGAGATTCCAGCTGCCTGGACTCCGCCTTCCCGGTTAGGCCACACACTATCGGTTTATGGAGGAAGAAAGATCTTGATGTTTGGTGGTCTTGCTAAGAGCGGGCCTTTGAAATT 720
ZTLα        GTGGAGAGAGATTCCAGCTGCCTGGACTCCGCCTTCCCGGTTAGGCCACACACTATCGGTTTATGGAGGAAGAAAGATCTTGATGTTTGGTGGTCTTGCTAAGAGCGGGCCTTTGAAATT 720
ZTLβ        GTGGAGAGAGATTCCAGCTGCCTGGACTCCGCCTTCCCGGTTAGGCCACACACTATCGGTTTATGGAGGAAGAAAGATCTTGATGTTTGGTGGTCTTGCTAAGAGCGGGCCTTTGAAATT 720

ZTL gDNA    CCGTTCGAGTGATGTCTTCACAATGGATCTAAGCGAAGAGGAGCCTTGTTGGAGGTGTGTGACCGGTAGCGGAATGCCTGGAGCAGGAAACCCAGGAGGAGTAGCACCACCACCAAGGCT 840
ZTLα        CCGTTCGAGTGATGTCTTCACAATGGATCTAAGCGAAGAGGAGCCTTGTTGGAGGTGTGTGACCGGTAGCGGAATGCCTGGAGCAGGAAACCCAGGAGGAGTAGCACCACCACCAAGGCT 840
ZTLβ        CCGTTCGAGTGATGTCTTCACAATGGATCTAAGCGAAGAGGAGCCTTGTTGGAGGTGTGTGACCGGTAGCGGAATGCCTGGAGCAGGAAACCCAGGAGGAGTAGCACCACCACCAAGGCT 840

ZTL gDNA    AGATCACGTTGCAGTTAACCTCCCTGGAGGCAGAATCTTGATATTTGGCGGCTCAGTGGCAGGGCTTCACTCAGCGTCTCAGCTTTATCTACTTGACCCAACGGAGGACAAACCGACTTG 960
ZTLα        AGATCACGTTGCAGTTAACCTCCCTGGAGGCAGAATCTTGATATTTGGCGGCTCAGTGGCAGGGCTTCACTCAGCGTCTCAGCTTTATCTACTTGACCCAACGGAGGACAAACCGACTTG 960
ZTLβ        AGATCACGTTGCAGTTAACCTCCCTGGAGGCAGAATCTTGATATTTGGCGGCTCAGTGGCAGGGCTTCACTCAGCGTCTCAGCTTTATCTACTTGACCCAACGGAGGACAAACCGACTTG 960

ZTL gDNA    GAGAATACTAAACATTCCAGGAAGACCGCCTCGGTTTGCTTGGGGACATGGCACTTGTGTTGTGGGAGGAACACGAGCGATAGTGCTAGGTGGTCAGACCGGAGAAGAATGGATGCTAAG 1080
ZTLα        GAGAATACTAAACATTCCAGGAAGACCGCCTCGGTTTGCTTGGGGACATGGCACTTGTGTTGTGGGAGGAACACGAGCGATAGTGCTAGGTGGTCAGACCGGAGAAGAATGGATGCTAA- 1079
ZTLβ        GAGAATACTAAACATTCCAGGAAGACCGCCTCGGTTTGCTTGGGGACATGGCACTTGTGTTGTGGGAGGAACACGAGCGATAGTGCTAGGTGGTCAGACCGGAGAAGAATGGATGCTAAG 1080

                                                                                                      Intron 2
ZTL gDNA    TGAGCTACACGAATTATCACTAGCGAGCTATCTCACGTAACCAAAGAGAGCCTATGTATGAAAGAGAACCCAGCCATAGAAGAAGATGAAAAAGGGTTCTCGGTTAGGATACTGGAGCTT 1200
ZTLa        -----------------------------------------------------------------------------------------------------------GATACTGGAGCTT 1092
ZTLβ        TGAGCTACACGAATTATCACTAGCGAGCTATCTCACGTAACCAAAGAGAGCCTATGTATGAAAGAGAACCCAGCCATAGAAGAAGATGAAAAAGGGTTCTCGGTTAGGATACTGGAGCTT 1200
                    Retention of intron 2              *                                                3'-UTR
                                                                      Exon 3
ZTL gDNA    TAGGGGAGAGCGACTCAGTGGTGGGACTTTGGTACTGCTCATATTTTTTAAGTCCTTCTTCTTCTTTCTTCCTCA 1275
ZTLa        TAGGGGAGAGCGACTCAGTGGTGGGACTTTGGTACTGCTCATATTTTTTAAGTCCTTCTTCTTCTTTCTTCCTCA 1167
ZTLβ        TAGGGGAGAGCGACTCAGTGGTGGGACTTTGGTACTGCTCATATTTTTTAAGTCCTTCTTCTTCTTTCTTCCTCA 1275
```

Additional file 5. Nucleotide sequence comparison of *ZTL* gDNA and *ZTLα* and *ZTLβ* cDNAs. The nucleotide sequences of *ZTLα* and *ZTLβ* cDNAs were determined by DNA sequencing of RT-PCR products and aligned with *ZTL* gDNA using the ClustalW software. Part of the aligned sequences containing exons 2 and 3 and intron 2 was displayed. Intron 2, which is retained in the *ZTLβ* transcript as a result of alternative splicing, is underlined (blue). A PTC is introduced into the *ZTLβ* transcript (red asterisk). The 3' untranslated region of the *ZTLβ* transcript is shown in gray.
