# Supplementary material for: Alternative splicing and nonsense-mediated decay of circadian clock genes under environmental stress conditions in Arabidopsis
Source: BMC Plant Biol. 2014 May 19;14:136. doi: 10.1186/1471-2229-14-136 (PMC4035800; doi:10.1186/1471-2229-14-136)
Supplement: Additional file 6 — The fate of TOC1β and ELF3β transcripts under heat stress conditions. Ten-day-old Col-0 plants and upf1-5 and upf3-1 mutants grown on ½ X Murashige & Skoog media containing 0.6% (w/v) agar plates (hereafter referred to as MS-agar plates) were transferred to 37°C for 12 h before harvesting whole plant materials for the extraction of total RNA. Levels of TOC1β and ELF3β transcripts were determined by quantitative real-time RT-PCR (qRT-PCR). Biological triplicates were averaged and statistically treated using Student t-test (*P<0.01). Bars indicate standard error of the mean. [file 1471-2229-14-136-S6.pdf]

Additional file 6

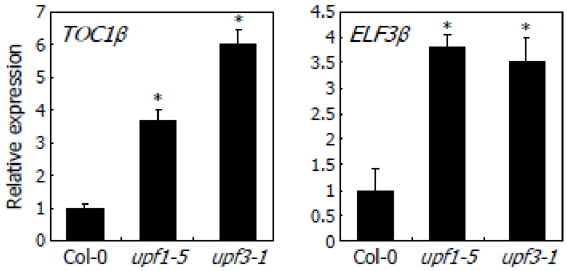

Additional file 6. The fate of *TOC1β* and *ELF3β* transcripts under heat stress conditions. Ten-day-old Col-0 plants and *upf1-5* and *upf3-1* mutants grown on ½ X Murashige & Skoog media containing 0.6% (w/v) agar plates (hereafter referred to as MS-agar plates) were transferred to 37°C for 12 h before harvesting whole plant materials for the extraction of total RNA. Levels of *TOC1β* and *ELF3β* transcripts were determined by quantitative real-time RT-PCR (qRT-PCR). Biological triplicates were averaged and statistically treated using Student *t*-test (*P<0.01). Bars indicate standard error of the mean.
