# Supplementary material for: Alternative splicing and nonsense-mediated decay of circadian clock genes under environmental stress conditions in Arabidopsis
Source: BMC Plant Biol. 2014 May 19;14:136. doi: 10.1186/1471-2229-14-136 (PMC4035800; doi:10.1186/1471-2229-14-136)
Supplement: Additional file 7 — The fate of ZTLα transcript. Plants were grown on MS-agar plates for 10 days under normal growth conditions. Ten-day-old Col-0 plants were transferred to liquid MS culture containing 20 μM cycloheximide (CHX). Following vacuum infiltration for 10 min, the plants were incubated for 5 h at 23°C under normal growth conditions before harvesting whole plant materials for the extraction of total RNA (left panel). The upf1-5 and upf3-1 mutants were not treated with CHX (right panel). Levels of ZTLα transcript were determined by qRT-PCR Biological triplicates were averaged. Bars indicate standard error of the mean. [file 1471-2229-14-136-S7.pdf]

Additional file 7

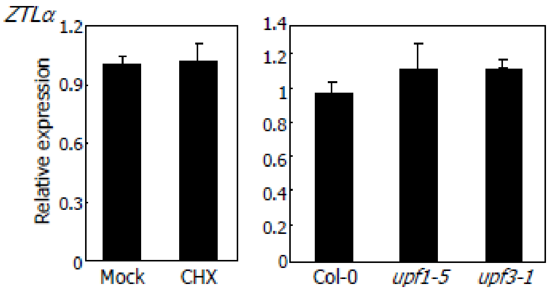

Additional file 7. The fate of *ZTLα* transcript.
Plants were grown on MS-agar plates for 10 days under normal growth conditions. Ten-day-old Col-0 plants were transferred to liquid MS culture containing 20 μM cycloheximide (CHX). Following vacuum infiltration for 10 min, the plants were incubated for 5 h at 23°C under normal growth conditions before harvesting whole plant materials for the extraction of total RNA (left panel). The *upf1-5* and *upf3-1* mutants were not treated with CHX (right panel). Levels of *ZTLα* transcript were determined by qRT-PCR Biological triplicates were averaged. Bars indicate standard error of the mean.
