# Supplementary material for: Alternative splicing and nonsense-mediated decay of circadian clock genes under environmental stress conditions in Arabidopsis
Source: BMC Plant Biol. 2014 May 19;14:136. doi: 10.1186/1471-2229-14-136 (PMC4035800; doi:10.1186/1471-2229-14-136)
Supplement: Additional file 8 — Primers used in qRT-PCR, RT-PCR, and gene cloning. F, forward primer; R, reverse primer. [file 1471-2229-14-136-S8.pdf]

## Additional file 8

| Primer | Usage | Sequence |
|---|---|---|
| eIF4a-F | qRT-PCR, RT-PCR | 5′-TGACCACACAGTCTCTGCAA |
| eIF4a-R | qRT-PCR, RT-PCR | 5′-ACCAGGGAGACTTGTTGGAC |
| CCA1a-F1 | qRT-PCR, RT-PCR | 5′-GATCTGGTTATTAAGACTCGGAAGCCATATAC |
| CCA1a-R1 | qRT-PCR, RT-PCR | 5′-GCCTCTTTCTCTACCTTGGAGA |
| CCA1b-F2 | qRT-PCR, RT-PCR | 5′-GAATGTTCCTTGTGATAAGCCATAGAGG |
| CCA1b-R2 | qRT-PCR, RT-PCR | 5′-AGGATCGTTCCACTTCCCGTCTT |
| PRR7a-F1 | qRT-PCR, RT-PCR | 5′-CCCTGTCATCATGATGTCATCTC |
| PRR7b-F2 | qRT-PCR, RT-PCR | 5′-CTAGCGTATGTTGGAAAGTAGATGC |
| PRR7-R | qRT-PCR, RT-PCR | 5′-TAGCATTCAGGCCAATGCTC |
| PRR9a-F1 | qRT-PCR, RT-PCR | 5′-CATTCCTGTCATAATGATGTCTTCTC |
| PRR9b-F2 | qRT-PCR, RT-PCR | 5′-CTGTCATAAGTACGATGTGATGTCTTC |
| PRR9-R | qRT-PCR, RT-PCR | 5′-GTCAATTCCACCAATCAAATCC |
| TOC1a-F1 | qRT-PCR, RT-PCR | 5′-AGGAAAATGAGTGGTCTGTTGC |
| TOC1b-F2 | qRT-PCR, RT-PCR | 5′-GTATTTGACAGTTTAAAGCCCTCTG |
| TOC1-R | qRT-PCR, RT-PCR | 5′-CGACAAGAGGAGGATCCTGAC |
| ELF3a-F1 | qRT-PCR, RT-PCR | 5′-CCATTGCCAATCAACAAAGAG |
| ELF3a-R1 | qRT-PCR, RT-PCR | 5′-TGGTCAGTCTTCTCCGAGTCAC |
| ELF3b-F2 | qRT-PCR, RT-PCR | 5′-CCATTGCCAACAGGGAGG |
| ELF3b-R2 | qRT-PCR, RT-PCR | 5′-AATACTCTTTGTTGACTGGTTCAACC |
| ZTL-F | qRT-PCR, RT-PCR | 5′-ACGTTGCAGTTAACCTCCCTG |
| ZTLa-R1 | qRT-PCR, RT-PCR | 5′-GCTCCAGTATCTTAGCATCCATTC |
| ZTLb-R2 | qRT-PCR, RT-PCR | 5′-TTACGTGAGATAGCTCGCTAGTGA |
| | | |
| CCA1a-AD-F | Cloning | 5′-CGGAATTCTATAGTGGCTGAGATTTCTCCATTTC |
| CCA1a-AD-R | Cloning | 5′-AAGGATCCTTTCATGTGGAAGCTTGAGTTTCCAA |
| CCA1b-AD-F | Cloning | 5′-CGGAATTCGAATGTTCCTTGTGATAAGCCATAGA |
| CCA1b-AD-R | Cloning | 5′-AAGGATCCTTTCATGTGGAAGCTTGAGTTTCCAA |
| PRR7a-AD-F | Cloning | 5′-CGGAATTCCCCTGTCATCATGATGTCATCTC |
| PRR7a-AD-R | Cloning | 5′-GGCCCGGGTTAGCTATCCTCAATGTTTTTTATGTC |
| PRR7b-AD-F | Cloning | 5′-CGGAATTCCTAGCGTATGTTGGAAAGTAGATGC |
| PRR7b-AD-R | Cloning | 5′-GGCCCGGGTTAGCTATCCTCAATGTTTTTTATGTC |
| PRR9a-AD-F | Cloning | 5′-GGAATTCCATATGCATTCCTGTCATAATGATGTCTTCTC |
| PRR9a-AD-R | Cloning | 5′-GGGGATCCTCATGATTTTGTAGACGCGTCTG |
| PRR9b-AD-F | Cloning | 5′-GGAATTCCATATGCTGTCATAAGTACGATGTGATGTCTTC |
| PRR9b-AD-R | Cloning | 5′-GGGGATCCTCATGATTTTGTAGACGCGTCTG |
| TOC1a-AD-F | Cloning | 5′-CGGAATTCAGGAAAATGAGTGGTCTGTTGC |
| TOC1a-AD-R | Cloning | 5′-CCGCTCGAGCTCAAGTTCCCAAA |
| TOC1b-AD-F | Cloning | 5′-CGGAATTCGTATTTGACAGTTTAAAGCCCTCTG |
| TOC1b-AD-R | Cloning | 5′-CCGCTCGAGCTCAAGTTCCCAAA |
| ELF3a, b-AD-F | Cloning | 5′-TCCCCCCGGGCCATGAAGAGAGGGAAAGATGAGGAG |
| ELF3a, b-AD-R | Cloning | 5′-CGGGATCCTTAAGGCTTAGAGGAGTCATAGCGT |
| ZTLa-AD-F | Cloning | 5′-CGGGATCCGCATGGAGTGGGACAGTGGTTC |
| ZTLa-AD-R | Cloning | 5′-CCGCTCGAGGCTCCAGTATCTTAGCATCCATTCTTCT |
| ZTLb-AD-F | Cloning | 5′-CGGGATCCGCATGGAGTGGGACAGTGGTTC |
| ZTLb-AD-R | Cloning | 5′-CCGCTCGAGTTACGTGAGATAGCTCGCTAGTG |

**Additional file 8. Primers used in qRT-PCR, RT-PCR, and gene cloning.**
F, forward primer; R, reverse primer.
